# Modification of a GnRH‐based system to synchronise oestrus in *Bos indicus* cattle improves pregnancy rates to AI in heifers but not cows

**DOI:** 10.1111/avj.13142

**Published:** 2022-02-02

**Authors:** J Cavalieri, S Das

**Affiliations:** ^1^ College of Public Health, Medical and Veterinary Science, James Cook University Townsville Queensland 4811 Australia; ^2^ College of Science and Engineering James Cook University Townsville Queensland 4811 Australia

**Keywords:** AI, *Bos indicus*, GnRH, oestrous synchronisation

## Abstract

**Objective:**

To investigate if modification of a gonadotrophin‐releasing hormone (GnRH) based protocol to synchronise oestrus in *Bos indicus* cattle will improve response rates to the first administration of GnRH and improve pregnancy rates to artificial insemination (AI).

**Design:**

Randomised control study.

**Methods:**

*Bos indicus* heifers, nonlactating and lactating cows allocated to a GnRH‐18 treatment (n = 237) were treated with an intravaginal device (IVD) and cloprostenol (0.5 mg IM) on day −11 and on day 0 remaining animals in the GnRH‐7 treatment (n = 222) were administered an IVD and every animal was treated with GnRH (100 μg IM). On day 7, equine chorionic gonadotrophin (400 IU IM) and cloprostenol were administered and IVD's were removed. Animals detected in oestrus on day 9 were artificially inseminated while those not detected in oestrus were administered GnRH (100 μg IM) at 1700 hours and inseminated on day 10. Bulls were inserted 2 weeks after completion of AI and remained until day 65.

**Results:**

The GnRH‐18 protocol increased the diameter of the largest follicle in the ovary on day 0, increased the percentage of new CL's induced after day 0 (46.3% vs 36.1%, for GnRH‐18 and GnRH‐7; P = 0.022), decreased circulating concentrations of progesterone on day 7 and increased odds of pregnancy to AI in heifers but not in nonlactating and lactating cows.

**Conclusion:**

Treatment with the GnRH‐18 compared to the GnRH‐7 protocol increased pregnancy rates to AI in heifers but not in nonlactating or lactating cows.

**Abbreviations**
AI: artificial insemination
ANOVA: analysis of variance
CL: corpus luteum
eCG: equine chorionic gonadotrophin
GnRH: gonadotrophin‐releasing hormone
IVD: intravaginal device
LH: luteinising hormone

Artificial insemination (AI) is used to promote genetic progress and alter the sex ratio of offspring in both beef and dairy cattle herds.^1^ In extensively managed beef herds, widespread use of AI is hampered by variable pregnancy rates to AI and relatively high labour and economic costs associated with applying treatments to synchronise oestrus and undertake AI. Synchronisation of oestrous cycles affords some efficiencies in time and labour enabling AI to be undertaken at set times limiting the period over which detection of oestrus occurs.^2^ A range of protocols have been used to synchronise oestrus in cattle.^3^, ^4^ Treatments that promote the most precise synchrony of oestrus and optimal fertility when timed AI is used include a treatment to induce a synchronous emergence of a new follicular wave and protocols that manage the duration of follicular dominance prior to ovulation so that oocytes are sufficiently developed but not too old when ovulation occurs.^5^

Administration of esters of oestradiol in conjunction with progesterone or administration of gonadotrophin‐releasing hormone (GnRH), with and without concurrent treatment with progesterone, have been the main hormones administered to synchronise new wave emergence just before AI.^4^, ^6^ When oestradiol is administered with progesterone, it induces atresia of existing dominant and emerging follicles through suppression of gonadotrophins resulting in synchronous new wave emergence in about 4 days.^7^ Administration of GnRH induces ovulation of dominant follicles, through induction of a surge release of LH (luteinising hormone), resulting in new wave emergence in about 2 days.^8^, ^9^

Administration of 17β‐oestradiol and its related esters to cattle for the purpose of oestrous synchronisation are currently banned by the European Union.^10^ Protocols using GnRH to regulate follicular development have been used as an alternative to the use of esters of oestradiol. Fertility to a timed AI can be adversely affected by variable ovulation rates following administration of GnRH at the start of protocols, resulting in failure to consistently induce new wave emergence.^2^, ^11^ This can lead to poor synchrony of ovulation following administration of GnRH during a synchronised pro‐oestrus^12^ and reduce fertility to a timed AI.^13^ Responses to administration of a first treatment with GnRH are generally greater when it is administered between days 5 and 12 of the oestrous cycle.^14^, ^15^ This is thought to be due to the requirement for follicles to be mature enough to have a sufficient number of LH receptors present to ovulate in the presence of an LH surge and this usually requires follicles to be ≥10 mm in diameter.^16^, ^17^ Presynchronisation of oestrous cycles prior to treating with GnRH has been used to attempt to increase the diameter of dominant follicles at the time of administering GnRH and improve responses to treatment.^13^, ^18^, ^19^, ^20^, ^21^ While such treatments have demonstrated improved pregnancy rates in some studies, two to three interventions are required with some presynchronisation protocols before commencing a GnRH‐based protocol making them less applicable in extensively managed beef herds. Use of AI on detection of oestrus or split‐timed AI are other strategies that have been used to help optimise pregnancy rates when variation in the pattern of onset of oestrus may reduce pregnancy rates to a single timed AI.^22^, ^23^ Split‐time AI involves inseminating females first detected in oestrus following administration of a treatment protocol to synchronise oestrus and then inseminating remaining animals at a set time the following day.

Lesser concentrations of progesterone during follicular wave emergence are associated with greater frequency of release of LH from the anterior pituitary gland and greater diameters of dominant follicles.^24^ Modification of a GnRH protocol by exposing females to lower concentrations of progesterone should favour the development of larger ovarian follicles before the time of administration of GnRH and may provide an opportunity to increase ovulation rates to the first treatment with GnRH and increase fertility to AI. Designing a treatment that minimises the number of interventions required prior to the first treatment with GnRH would also reduce requirements for labour for a presynchronisation treatment.

The aim of this study was to develop a single‐step, presynchronisation treatment that would help improve ovulatory responses to the first GnRH treatment in *Bos indicus* females. Our hypothesis was that pretreatment of *B. indicus* females with a low dose of progesterone using an intravaginal progesterone releasing device before administration of GnRH will increase the diameter of the largest ovarian follicle present at the time of administration of GnRH, increase the odds of inducing a new CL (corpus luteum) after administering GnRH and improve the synchrony of oestrus and fertility when using a split‐timed AI protocol.

## Materials and methods

### Experimental site

This study was conducted between 2016 and 2018 across three successive breeding seasons at the James Cook Tropical Veterinary Research station, Fletcherview, which is located in a dry tropical region of northern Queensland (latitude 19°53′4″S; longitude 146°10′43″E). Cattle grazed native pastures and improved pastures consisting predominantly of *Cenchrus cillaris* (Buffel grass). Ethics approval to conduct the study was provided by the James Cook University Experimentation Ethics Review Committee (approval number: A2247).

### Animals and treatments

**Figure 1:**
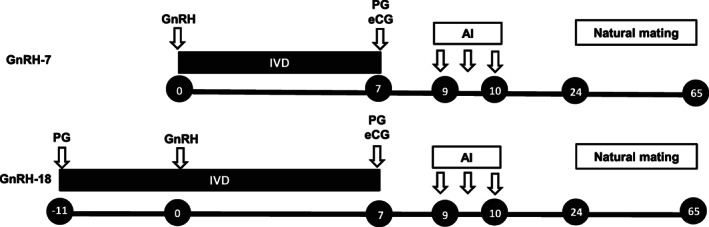
Outline of the treatment protocol. *Bos indicus* nulliparous heifers (n = 165), nonlactating (n = 102) and lactating cows (n = 192) were weighed, body conditions scored (1–9 scale) and examined with transrectal ultrasonography on day −11 to determine the presence or absence of a visible CL and allocated to one of two treatments. Animals allocated to the GnRH‐18 treatment were administered an intravaginal progesterone releasing device (IVD) containing 1.56 g of progesterone and cloprostenol (PG; 0.50 mg, IM) on day −11. On day 0 animals were re‐examined using transrectal ultrasonography and an IVD was administered to the remaining animals allocated to the GnRH‐7 treatment. Every animal was treated with GnRH (100 μg IM) on day 0. On day 7, animals were re‐examined with transrectal ultrasound and eCG (400 IU IM) and PG (0.5 mg IM) was administered, IVDs removed and rump mounted oestrous detection aids were applied. Animals detected in oestrus on day 9 in the morning and afternoon were artificially inseminated. Animals not detected in oestrus were administered GnRH (100 μg IM) in the afternoon (1700 hours) and inseminated on day 10. Bulls were placed with the herd 2 weeks after completion of AI and remained with animals until at least day 65.

*Bos indicus* (Brahman) nulliparous heifers (1–3 years, n = 165), nonlactating (3–11 years, n = 102) and lactating cows (3–11 years, n = 192) were enrolled in the study. Animals were weighed, body conditions scored where 1 = emaciated and 9 = obese^25^ and had their reproductive tracts examined with transrectal ultrasonography on day −11 to determine the presence or absence of a visible CL. Animals (n = 459) were stratified by age and animal type (heifers, nonlactating cows and lactating cows) and then randomly allocated to one of two treatments (Figure 1). Animals allocated to the 18‐day GnRH treatment were administered an intravaginal progesterone releasing insert (intravaginal device – IVD; Cue‐mate, Vetoquinol, Da Vinci Business Park Brisbane Airport, QLD, Aust) containing 1.56 g of progesterone on day −11 and cloprostenol (0.50 mg, IM; Juramate; Jurox, Rutherford, NSW, Aust) was administered. Eleven days later (day 0) animals were re‐examined using transrectal ultrasonography and an identical intravaginal insert that was administered to animals on day −11 was administered to the remaining animals allocated to the 7‐day GnRH treatment. Every animal was also treated with GnRH (100 μg IM, gonadorelin acetate, Ovurelin, Bayer Australia, Pymble, NSW, Aust) on day 0. Seven days later animals were re‐examined with transrectal ultrasound and equine chorionic gonadotrophin (eCG; 400 IU IM; Pregnecol, Vetoquinol) and cloprostenol (0.5 mg IM) were administered and IVDs were removed. Rump mounted oestrous detection aids (Estrotect, Genetics Australia, Bacchus Marsh, VIC, Aust) were also applied. Forty‐eight hours after removal of inserts (day 9) animals were examined and those detected in oestrus were artificially inseminated. Animals were re‐examined 6–8 h later and those detected in oestrus were inseminated while those not detected in oestrus were administered GnRH (100 μg IM) at about 1700 hours and inseminated on day 10, about 72 h after removing IVDs. Bulls were placed with the herd 2 weeks after completion of AI at a mating ratio of about 2.5% in accordance with normal management practices for the herd and were removed between 56 and 66 days after first AI.

### Artificial insemination

Animals were artificially inseminated by the same veterinarian throughout the course of the study using either frozen–thawed semen from six sires or chilled semen from one sire. Chilled semen was prepared by extending semen collected using an electroejaculator (Lane Pulsator IV, Lane Manufacturing Inc. Denver, CO, USA) with a commercial extender (Andromed, Minitube Australia, Smythesdale, VIC, Aust) ensuring there were at least 10 million progressively motile and normal sperm per dose. Chilled semen was stored at 5°C and used for AI within 40 h of collection.

### Detection of oestrus

Animals were assessed as being in oestrus or not at 48, 54 and 72 h after removal of inserts based on the proportion of the background colour of aids for the detection of oestrus that was visible. Aids for the detection of oestrus were assigned the following scores: 0 = detector lost or 100% background colour visible, 1 = ≤100% to 75% visible, 2 = <75% to 50% visible, 3 = <50% to 25% visible, 4 = <25% to none of the background colour being visible. Cows that were observed to stand while being mounted and cows with a score of ≤3 were classified as being in oestrus.

### Ultrasonography

Transrectal ultrasonography was performed on each cow using a 7.5 MHz, linear transducer (Mylab 5; Medical Plus Australia Pty Ltd, Tullamarine, VIC, Aust) on days −18, 0, 7 and on a random selection of animal at the time of AI in 2018 (n = 69). A video recording of each ultrasound examination was made and subsequently the number of follicles ≥3.0 mm, the diameter (maximum length and width/2) and location of the largest and second‐largest follicle and any corpora lutea were recorded on hand‐drawn ovarian maps. Ovulation following administration of GnRH on day 0 was determined by assessing the location, presence or absence and the number of corpora lutea observed on days 0 and 7. Corpora lutea were recorded as being induced when no CL was observed to be present in an ovary on day 0 but a CL was visible in the same ovary on day 7 or if a second CL was visible on day 7 in an ovary when only one was observed in the same ovary on day 0.^26^ Pregnancy diagnosis was also undertaken with the aid of the same transrectal ultrasonography unit between 12 and 14 weeks after first AI and used to estimate gestation length.^27^, ^28^

### Progesterone assay

Blood samples were collected from the coccygeal vein or artery into plain evacuated tubes, from a randomly selected group of animals in both treatment groups on day 7 at the time of removal of inserts in 2016. A total of 16 heifers, 6 nonlactating cows and 39 lactating cows were sampled in the GnRH‐7 treatment and 22 heifers, 6 nonlactating cows and 37 lactating cows were sampled in the GnRH‐18 treatment. Blood samples were stored at room temperature for 1 h then placed on ice and centrifuged at 3000 *g* within 4 h of collection. Serum was then separated and stored at −20°C until the time of assay. Concentrations of progesterone were measured in duplicate using a solid‐phase radioimmunoassay kit (IM1188, Immunotech, Prague, Czech Republic).^29^, ^30^ The sensitivity of the assay was 0.09 ng/mL. The intra‐ and inter‐assay variation for a plasma pool of 2.99 ± 0.08 ng/mL was 4.6% and 8.2%, respectively, and for a plasma pool of 13.89 ± 0.44 ng/mL was 4.7% and 7.7%, respectively.

### Statistical analyses

Statistical analyses were performed using the statistical software IBM SPSS version 25 (SPSS Inc., Chicago, IL, USA). Mean diameters of the largest ovarian follicle measured in the ovaries on days 0 and 7 were compared using analysis of variance (ANOVA). Factors included in the models were year, animal type (heifer, nonlactating cow and lactating cow) and the treatment by type interaction. Year was dropped after initial analyses as the effect of year was not significant (P > 0.25). The mean diameter of the largest follicle present at the time of AI in animals in 2018 was also compared using ANOVA and included effects due to treatment and animal classification and their interaction.

Multivariable logistic regression was used to determine the odds of inducing a new CL after administration of GnRH on day 0, the odds that oestrus would be detected at 48, 54 and 72 h after removal of IVDs and pregnancy rates to AI and 8 weeks after the first AI. Factors considered in models included treatment, year, type (heifer or nonlactating cow, lactating cow), body condition score (≤3, 3.5 to 5.5, ≥6), whether animals had a CL visible in ovaries at the start of treatment and weight at the start of the study (day −11) and sire and relevant interactions. Terms were considered for elimination from each model using backward stepwise logistic regression although treatment was retained in every model. The test for elimination was the Wald Chi‐Square statistic using a significance level of P ≥ 0.10. If an interaction was significant at P < 0.10, the associated main effects were included in the model. Goodness of fit of the models was assessed using the Hosmer and Lemeshow test. Probability values for all main effects remaining in models were determined using the approximate Chi‐Squared distribution of the Wald statistic. Odds ratios and 95% confidence intervals were also calculated for all main effects remaining in models. Mean concentrations of progesterone on day 7 were compared with ANOVA with treatment, animal type and their interaction included in the model.

## Results

### Exclusions

Three animals were inseminated at 72 h after removal of IVDs but the oestrous status of the animals was not recorded. These animals were excluded from analysis of the percentage of animals in oestrus at the time of AI at 72 h after removal of inserts.

Incomplete recording of ultrasound examinations occurred with 12 females (4 heifers, 1 nonlactating cow and 7 lactating cows). Data were not included in the percentage of animals that had a CL on day 0 (n = 2) and whether a CL was induced or not (n = 12).

### Pattern of onset of oestrus

**Figure 2:**
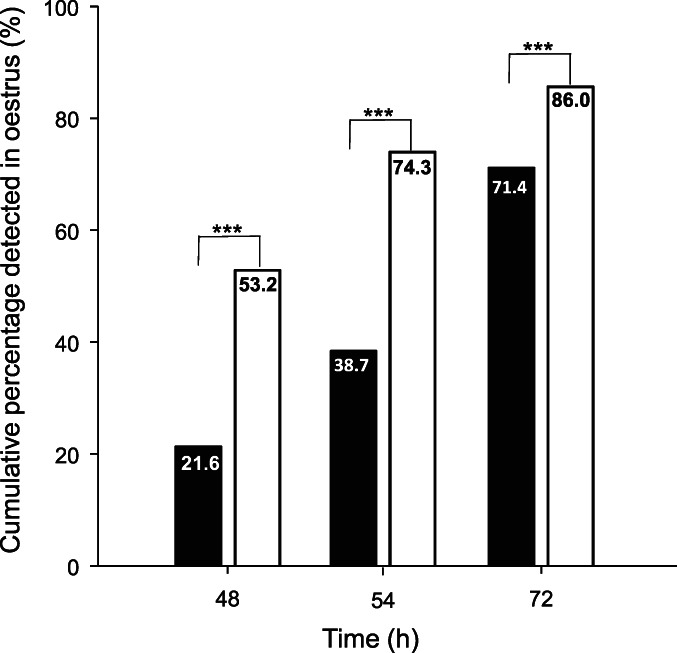
Cumulative percentage of females detected in oestrus at 48, 54 and 72 h after removal of intravaginal progesterone releasing devices. GnRH‐7 females (■), GnRH‐18 females (□).

The cumulative percentage of animals classified as being in oestrus at 48, 54 and 72 h after removal of inserts was significantly less in the animals treated with the GnRH‐7 compared to the GnRH‐18 protocol (Figure 2). The odds of animals being detected in oestrus by 48 and 72 h varied between years and by 72 h after removal of inserts animals with body condition score (BCS) between 3 and 6 tended to have lesser odds of being detected in oestrus than those with a BCS ≤3 (P = 0.069) and greater odds of being detected in oestrus compared to those with a BCS ≥6 (P = 0.094; Table 1).

**Table 1:** Analysis of odds ratio estimates for effects of treatment on cumulative percentages of animals detected in oestrus after removal of intravaginal devices with a 7‐day (GnRH‐7) or 18‐day (GnRH‐18) treatment protocol

Interval (h)	Covariate	df	B	SE	χ^2^	Odds ratio	95% CI	*P*‐value^a^	Reference group
48	GnRH‐7	1	−1.49	0.22	47.1	0.23	0.15–0.35	<0.001	GnRH‐18
	Heifers	1	−0.02	0.25	0.006	0.98	0.61–1.59	0.938	Lactating cows
	Nonlactating cows	1	0.51	0.28	3.35	1.66	0.97–2.87	0.067	Lactating cows
	Year 1	1	0.62	0.29	4.47	1.85	1.05–3.27	0.035	Year 3
	Year 2	1	1.11	0.26	17.8	3.02	1.81–5.05	<0.001	Year 3
54	GnRH‐7	1	−1.52	0.20	56.1	0.22	0.15–0.33	<0.001	GnRH‐18
72	GnRH‐7	1	−0.98	0.25	15.5	0.37	0.23–0.61	<0.001	GnRH‐18
	Year 1	1	−2.93	0.74	15.9	0.05	0.01–0.23	<0.001	Year 3
	Year 2	1	−2.17	0.67	10.4	0.11	0.03–0.43	<0.001	Year 3
	BCS ≤3	1	0.72	0.40	3.30	2.06	0.94–4.48	0.069	3 < BCS < 6
	BCS ≥6	1	−1.13	0.67	2.81	0.32	0.09–1.21	0.094	3 < BCS < 6

B, co‐efficient; BCS, body condition score; CI, 95% confidence interval; df, degrees of freedom; SE, standard error.

^a^ Statistical significance is based on Wald‐Chi Square test statistic.

### Ovarian follicular development

**Figure 3:**
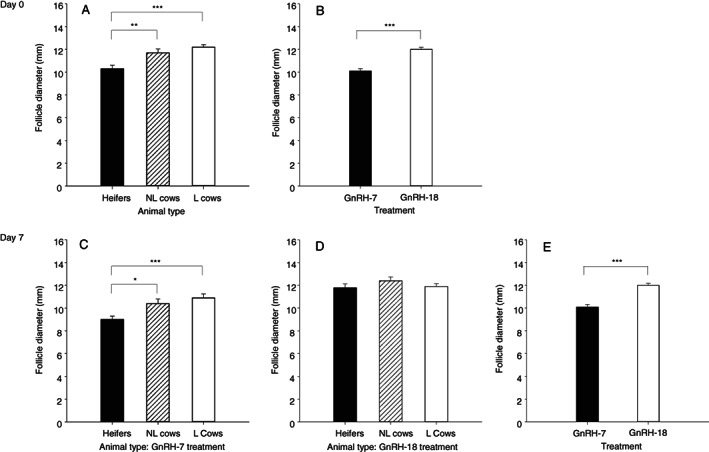
Mean diameter of the largest ovarian follicle on day 0 (A) and 7 (C, GnRH‐7 treated animals; D, GnRH‐18 treated animals) in heifers, nonlactating (NL) and lactating (L) cows and all animals treated with the GnRH‐7 and GnRH‐18 protocols on days 0 (B) and 7 (E).

The diameter of the largest follicle imaged in the ovaries on day 0 differed between treatments (P < 0.001) and with the type (heifer, nonlactating cow or lactating cow) of animal (P < 0.001) but the treatment by type interaction was not significant (P = 0.802). The mean diameter of the largest follicle was less in heifers compared to nonlactating and lactating cows on day 0 (P < 0.05; Figure 3A). The mean diameter of the largest follicle on day 0 was greater in the animals treated with GnRH‐18 compared to the GnRH‐7 protocol (P < 0.001; Figure 3B).

The diameter of the largest follicle imaged in ovaries on day 7 was affected by treatment (P < 0.001), the type of animal (P = 0.001) and there was a treatment by type interaction (P = 0.014). In animals administered the GnRH‐7 treatment, the mean diameter of the largest follicle on day 7 was less in the heifers compared to the nonlactating and lactating cows (P < 0.05; Figure 3C), but for animals administered the GnRH‐18 treatment, the diameter of the largest follicle on day 7 did not differ significantly between animal types (Figure 3D). The mean diameter of the largest follicle on day 7 was less in the animals treated with the GnRH‐7 compared to those treated with the GnRH‐18 protocol (P < 0.001; Figure 3E).

The mean diameter of the largest follicle imaged in the ovary at the time of AI did not differ between animals treated with the GnRH‐7 and GnRH‐18 protocols (12.3 ± 0.39 and 13.2 ± 0.58 mm, respectively; P = 0.339) or animal types (12.4 ± 0.42, 12.4 ± 0.69, 13.6 ± 0.85 mm, for the heifers, nonlactating and lactating cows, respectively; P = 0.380) and the interaction was not significant (P = 0.679). The mean diameter of the largest follicle at the time of AI was greater in animals in which a CL was not visible on day −11 compared to those in which one was visible (13.6 ± 0.05 vs 11.9 ± 0.43 mm, respectively; P = 0.012).

### Pregnancy rates

**Figure 4:**
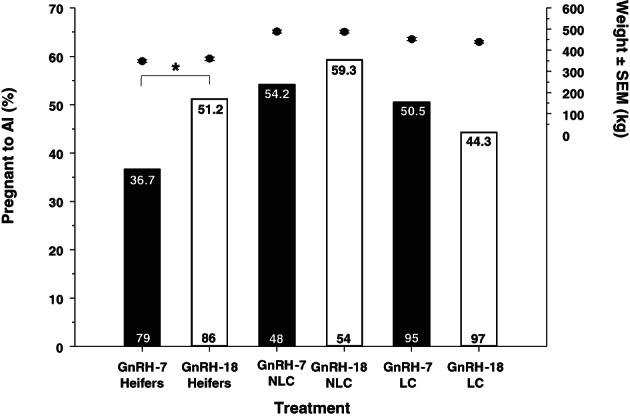
The mean ± SEM body weights (●) and pregnancy rates of heifers, nonlactating (NLC) and lactating (LC) cows treated with the GnRH‐7 or GnRH‐18 protocols to synchronise oestrus. Numbers at the base of bars represent numbers of animals. Percentages diagnosed pregnant to AI are presented at the top of bars.

Pregnancy rates obtained in different animal types and treatments across the 3 years of the study are listed in Table 2. The odds of animals being diagnosed as pregnant to AI was greater in the nonlactating compared to the lactating cows (P = 0.031) and tended to be increased in animals without a CL visualised on day −11 (P = 0.054; Table 3). The rate of change in the odds of pregnancy to AI was 57% lower (OR: 0.43, CI: 0.18–1.0; Table 3) in the heifers treated with the GnRH‐7 compared to the GnRH‐18 protocol (P = 0.049) but no significant interaction was found between treatment and the odds of pregnancy in the nonlactating and lactating cows (Figure 4). Further confirmation was found by assessing comparisons, using the same model covariates but repeating the analysis for each animal type separately. Separate analyses again confirmed a significant reduction in the odds of pregnancy for heifers but not lactating and lactating cows treated with the GnRH‐7 compared to the GnRH‐18 protocols (Table A1). Pregnancy rates obtained in each treatment and each type of animal along with their mean body weights are illustrated in Figure 4.

**Table 2:** Pregnancy rates of maiden heifers, nonlactating and lactating cows enrolled in an oestrous synchronisation study during the 3 years of the study and the percentage of corpora lutea induced following the first treatment with GnRH

Variable	Treatment						
	Year	GnRH‐7			GnRH‐18		
		Heifers	NL cows	Lactating cows	Heifers	NL cows	Lactating cows
Pregnancy rate to AI (%)	2016	29.4 (5/17)	33.3 (2/6)	56.4 (22/39)	50.0 (11/22)	42.9 (3/7)	46.2 (18/39)
	2017	33.3 (9/27)	60.7 (17/28)	45.7 (16/35)	42.9 (12/28)	65.5 (19/29)	42.5 (17/40)
	2018	42.9 (15/35)	50.0 (7/14)	47.6 (10/21)	58.3 (21/36)	55.6 (10/18)	44.4 (8/18)
	Total	36.7 (29/79)	54.2 (26/48)	50.5 (48/95)	51.2 (44/86)	59.3 (32/54)	44.3 (43/97)
8‐week pregnancy rate (%)	2016	35.3 (6/17)	66.7 (4/6)	76.9 (30/39)	59.1 (13/22)	71.4 (5/7)	53.8 (21/39)
	2017	74.1 (20/27)	96.4 (27/28)	82.9 (29/35)	71.4 (20/28)	96.6 (28/29)	67.5 (27/40)
	2018	85.7 (30/35)	78.6 (11/14)	71.4 (15/21)	88.9 (32/36)	88.9 (16/18)	77.8 (14/18)
	Total	70.9 (56/79)	87.5 (42/48)	77.9 (74/95)	75.6 (65/86)	90.7 (49/54)	63.9 (62/97)
CL induced (%)	Total	26.0 (20/77)	37.5 (18/48)	44.0 (40/91)	29.8 (25/84)	47.2 (25/53)	60.6 (57/94)

AI, artificial insemination; CL, corpus luteum; GnRH, gonadotrophin‐releasing hormone; NL, non‐lactating.

**Table 3:** Results of logistic regression analysis of factors affecting pregnancy rates to AI and cumulative pregnancy rates 8 weeks after commencing AI in animals treated with a 7‐ or 18‐day GnRH‐based treatment

Variable	Covariate	df	B	SE	χ^2^	Odds ratio	CI	P‐value	Reference group
Pregnancy rate to AI	GnRH‐7	1	0.22	0.29	0.57	1.25	0.70–2.21	0.450	GnRH‐18
	Heifers	1	0.40	0.31	1.68	1.49	0.82–2.71	0.197	Lactating cow
	NL cows	1	0.77	0.36	4.67	2.16	1.07–4.36	0.031	Lactating cow
	No CL visualised day −11	1	0.40	0.21	3.73	1.49	0.99–2.23	0.054	CL visualised day −11
	GnRH‐7 heifers	1	−0.85	0.43	3.88	0.43	0.18–1.00	0.049	GnRH‐18 heifers
	GnRH‐7 NL cows	1	−0.42	0.50	0.71	0.66	0.25–1.74	0.401	GnRH‐18 NL cows
8‐week pregnancy rate	GnRH‐7	1	0.72	0.33	4.69	2.06	1.07–3.96	0.030	GnRH‐18
	Heifers	1	0.40	0.34	1.36	1.49	0.76–2.90	0.243	Lactating cows
	NL cows	1	1.46	0.52	7.79	4.31	1.54–12.0	0.005	Lactating cows
	2016	1	−1.11	0.30	13.5	0.33	0.18–0.60	<0.001	2018
	2017	1	−0.23	0.30	0.56	0.80	0.44–1.44	0.453	2018
	GnRH‐7 heifers	1	−1.02	0.49	4.30	0.36	0.14–0.95	0.038	GnRH‐18 heifers
	GnRH‐7 NL cows	1	−1.06	0.73	2.13	0.35	0.08–1.44	0.144	GnRH‐18 NL cows
CL induced day 0	GnRH‐7	1	−0.46	0.20	5.26	0.63	0.43–0.94	0.022	GnRH‐18
	Heifers	1	−1.06	0.23	21.2	0.35	0.22–0.54	<0.001	Lactating cows
	NL cows	1	−0.41	0.25	2.66	0.66	0.41–1.09	0.103	Lactating cows

B, co‐efficient; CI, 95% confidence interval; df, degrees of freedom; NL, nonlactating; SE, standard error.

Odds of pregnancy, 8 weeks after the start of breeding were 4.3 times greater in the nonlactating compared to the lactating cows (89.2%, 91/102 vs 70.8%, 136/192, OR: 4.31, CI: 1.54–12.0; P = 0.005; Table 3). An interaction was detected between treatment and animal type with rate of change in the odds of pregnancy in animals being 51% less for heifers in the GnRH‐7 compared to the GnRH‐18 treatments (OR: 0.49, CI: 0.14–0.95; P = 0.038) but no significant interaction was found between treatment and the odds of pregnancy in the nonlactating cows (Table 3). Splitting the analysis by animal type revealed (2.02 times) greater odds of pregnancy in the lactating cows treated with the GnRH‐7 compared to GnR‐18 protocol (77.9%, 74/95 vs 63.9%, 62/97); OR: 2.02, CI: 1.06–3.83; P = 0.032; Table A1) but not between heifers (P = 0.352) and nonlactating cows (P = 0.510; Table A1). Nagelkerke's R^2^ value for the original model was 0.116 and for the model used to examine pregnancy rates after 8 weeks in the heifers alone was 0.164. This indicated that the model in which pregnancy rates in heifers after 8 weeks of breeding were examined separately for heifers more closely predicted the observed nonpregnant and pregnant outcomes slightly. The odds of pregnancy 8 weeks after the start of breeding were less in 2016 compared to 2018 (60.8%, 79/130 vs 83.1%, 118/142; OR: 0.33, CI: 0.18–0.60; P < 0.001; Table 3).

### Induction of corpora lutea after administration of GnRH

The odds of detecting a CL in ovaries with ultrasound on day 0 was greater in the animals that commenced treatment on day 0 (GnRH‐7 treatment) compared to those that commenced treatment on day −11 (GnRH‐18 treatment; 62.4%, 138/221 vs 19.9%, 47/236, respectively; P < 0.001). After administering GnRH on day 0, the odds of inducing a new CL by day 7 were less in the animals treated with the GnRH‐7 protocol compared to those treated with the GnRH‐18 protocol (36.1%,78/216 vs 46.3%, 107/231, respectively; Table 2; OR: 0.35; 95% CI: 0.22–0.54; P = 0.022, Table 3). It was also affected by the type of animal (P < 0.001; Table 3), being less in heifers compared to lactating cows but similar between nonlactating and lactating cows (28.0%, 45/161, 42.6%, 43/101, 52.4%, 97/185, for heifers, nonlactating and lactating cows, respectively). Effects of other variables and interactions were not significant.

**Figure 5:**
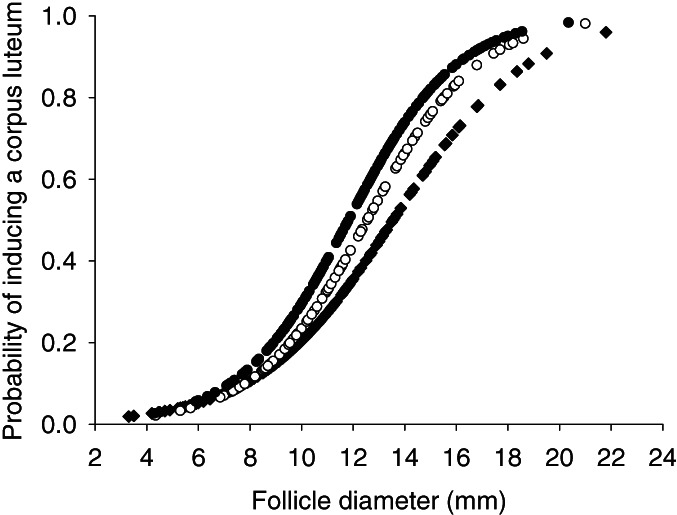
Fitted curves for the predicted probabilities of inducing a corpus luteum in *Bos indicus* females in relation to the diameter of the largest follicle (mm) imaged on the ovary at the time of administration of the first injection of GnRH on day 0. Heifers (■), nonlactating cows (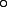), lactating cows (●).

The diameter of the largest follicle imaged in the ovaries on day 0 affected the probability of inducing a CL after administering GnRH on day 0 in the heifers, nonlactating and lactating cows (P < 0.001; Figure 5).

### Concentrations of progesterone

Concentrations of progesterone were transformed (Log10) to achieve homogeneity of variance. Mean concentrations of progesterone on day 7 were greater in the animals treated with the GnRH‐7 compared to the GnRH‐18 protocol (9.45 ± 0.58 vs 5.39 ± 0.45 ng/mL, respectively; P < 0.001). Effects of animal type (P = 0.431) and the interaction between type and treatment (P = 0.582) were not significant.

## Discussion

Administration of GnRH is used at the start of protocols to synchronise oestrus in cattle to help synchronise preovulatory follicular wave emergence, reduce variation in follicle maturity at a timed AI and thereby improve pregnancy rates to AI.^5^ In this study, we aimed to modify the treatment protocol in attempt to improve ovulation rates to the first treatment with GnRH and, as a result, improve the synchrony of preovulatory follicular wave emergence before AI and pregnancy rates to AI. The results of the study demonstrated that the use of an 18‐day modified protocol improved the odds of pregnancy to AI in heifers but not in nonlactating or lactating cows when using a split‐time AI strategy. While the initial treatment with GnRH significantly improved the number of new CL's induced by day 7 in the animals treated with the 18‐ compared to the 7‐day protocol, the percentage of new CL induced in heifers treated with the 18‐day protocol was still relatively low (29.8%) indicating that further modification of the protocol is needed to improve response rates to the first administration of GnRH in heifers.

The 18‐day protocol was designed to increase the diameter of the largest follicle in the ovary on day 0 in an attempt to increase the percentage of animals that would ovulate in response to the first treatment with GnRH. The modified treatment did significantly increase the mean diameter of the largest follicle in the ovary on day 0 and this was likely due to exposure to relatively low circulating concentrations of progesterone in the animals between day −11 and 0. Unfortunately, concentrations of progesterone in serum were not recorded on day 0 but administration of cloprostenol on day −11 did remove most functional corpora lutea as evidenced by a lesser percentage of corpora lutea being observed on day 0 in the GnRH‐18 compared to GnRH‐7 animals (19.9% vs 62.4%; P < 0.001). Treatment with an IVD for 11 days before administering the first treatment of GnRH would have also been expected to result in lower circulating concentrations of progesterone compared to animals in the GnRH‐7 treatment due to declining progesterone content within IVDs.^31^, ^32^ This also is the most likely reason, concentrations of progesterone were significantly less in the animals treated with the GnRH‐18 protocol on day 7. Lesser mean circulating concentrations of progesterone in the GnRH‐18 animals during treatment would have been expected to cause an increase in the frequency of release of LH from the anterior pituitary gland^24^ and explain the increase in the diameter of the largest follicle that was observed on day 0 and 7 in the GnRH‐18 compared to the GnRH‐7 treated animals. This same physiological reason could also explain why females without a CL observed in the ovaries on day −11 also tended to have greater odds of pregnancy to AI (P = 0.054, Table 3) and a larger follicle present at the time of AI (P = 0.012) due to lesser concentrations of progesterone during treatment resulting in more mature follicles at the time of AI.

Use of the modified treatment increased the percentage of animals in which a new CL was induced by 10.2% overall although only by 3.8% in heifers with the mean percentage of induced corpora lutea ranging between 26.0% in heifers in the GnRH‐7 treatment and 60.6% in the GnRH‐18 treated lactating cows (Table 2). Initial treatment with GnRH in cattle has resulted in ovulation rates of between 33% and 66%, which is similar to the range obtained across treatments and cattle types in this study, which recorded the percentage of new CL's induced as a measure of ovulation rates.^17^, ^18^, ^33^, ^34^, ^35^, ^36^ A relatively low rate of inducing a new CL after administering GnRH (14.3%) was recorded recently after administration of 100 μg or 250 μg of gonadorelin and an IVD in *B. indicus* females at the same experimental site (M Abdallah unpublished). In that study, the odds of inducing a new CL after administration of GnRH was found when animals lacked a CL at the time of insertion of an IVD and administration of GnRH, which would have contributed to the greater percentage of animals with a new CL induced in this study in animals treated with the GnRH‐18 protocol compared to that of M Abdallah (unpublished). Results from both studies also suggested that responses to administration of GnRH can be variable, which could compromise the ovulatory response at the end of the protocol and pregnancy rates in some animals after the initial administration of GnRH especially when undertaking a timed AI.

In this study, the odds of inducing a new CL after the first treatment with GnRH were less in heifers compared to nonlactating and lactating cows (28.0%, 45/161, 42.6%, 43/101, 52.4%, 97/185, respectively), which is similar to the observations of others who have recorded lower ovulation rates in heifers compared to cows following administration of GnRH to dairy^37^ and *B. indicus* cattle (M Abdallah unpublished). This has been attributed to an increased likelihood that a larger follicle with more LH receptors will be present in cows compared to heifers when administering GnRH (M Abdallah unpublished). Our analysis also revealed a greater predicted probability of inducing a new CL in cattle at the time of administering a first injection of GnRH when larger ovarian follicles were present. This finding is similar to previous findings reported in dairy cattle.^38^ In our study about a 70% probability of ovulation occurred when follicles were greater than about 16 mm in diameter in heifers. Our interpretation of the results suggest that if ovulation rates to administration of the initial injection of GnRH are to be increased that the maturity of dominant follicles present at the time of administering GnRH should be increased even further than was obtained on average on day 0 in this study and especially in heifers. This will likely require a longer duration of treatment with an IVD and/or administration of a lower dose of progesterone to induce greater frequency of release of LH and larger dominant follicles prior to administering GnRH.^24^

There was some evidence from the model used to examine the effects of treatment on 8‐week pregnancy rates that included all of the animal types that pregnancy rates were greater in the heifers treated with the GnRH‐18 compared to the GnRH‐7 protocol, however, pregnancy rates only differed by 4.7%. This was possibly due to the modest gains achieved at the time of AI in the heifers in the GnRH‐18 group. We did not, however, find a significant difference in 8‐week pregnancy rates when using the model that only included the heifers suggesting that differences in pregnancy rates after 8 weeks when using these treatments is relatively small or more statistical power would be needed with additional studies to determine the nature of the true effect of treatment with both protocols. Reasons for the 14% greater 8‐week cumulative pregnancy rate in the lactating cows treated with the GnRH‐7 compared to the GnRH‐18 protocol are unknown but reinforce the concept that there was no advantage in adopting the modified protocol in lactating cows.

In this study, greater odds of pregnancy to AI were recorded in heifers treated with the GnRH‐18 compared to the GnRH‐7 protocol in spite of no significant increase in the percentage of new CL's induced after treatment with GnRH on day 0. Mean concentrations of progesterone on day 7 were less in the animals treated with the GnRH‐18 compared to the GnRH‐7 protocol suggesting that animals treated with this protocol were exposed to lesser concentrations of progesterone during development of preovulatory follicles. Exposure to lesser concentrations of progesterone during preovulatory follicular development in *B. indicus* cattle before a timed AI has resulted in the development of larger ovarian follicles^39^, ^40^, ^41^, ^42^ and an increase in pregnancy rates in some^39^, ^40^, ^41^, ^43^ but not all studies.^44^, ^45^, ^46^ Greater circulating concentrations of progesterone during preovulatory follicular development enhanced pregnancy rates in anovulatory *B. indicus* females but sometimes decreased pregnancy rates to AI in females that were classified as ovulatory at the start of treatment with a synchronisation protocol.^42^ Lower circulating concentrations of progesterone during preovulatory follicular development in heifers treated with the GnRH‐18 protocol could have increased the probability that ovarian follicles had reached a more optimal stage of maturity at the time of AI in this study, which enhanced their pregnancy rates to AI. This is supported by our observation that treatment with the GnRH‐18 protocol increased the percentage of females that were detected in oestrus at 48, 54 and 72 h after removal of IVD's. In a metanalysis conducted using data from 10,116 beef females in 22 studies that examined results from five commonly used fixed‐time AI protocols, it was found that cows and heifers that were detected in oestrus before fixed‐time AI had a 27% greater (P < 0.05; 95% CI = 22%–32%) pregnancy rate to AI compared with those that were not detected in oestrus.^47^ Collectively, the improvement in pregnancy rates in the GnRH‐18 treated heifers may have been due to lower concentrations of progesterone during preovulatory follicular development that contributed to follicles reaching a more optimal stage of maturity at the time of AI and more heifers being in oestrus at the time of AI compared to the GnRH‐7 treated heifers.

## Conclusions

Use of a modified GnRH protocol in combination with administration of an IVD for 18 compared to 7 days increased the diameter of the largest follicle imaged in the ovary at the time of the first injection of GnRH and increased odds of pregnancy to AI in heifers but not in nonlactating and lactating *B. indicus* females. The modified treatment strategy increased the mean diameter of the largest follicle imaged in the ovary at the time of removing IVDs, the percentage of animals in oestrus at 48, 54 and 72 h after the removal of IVD's, increased the percentage of new CL's induced following the first administration of GnRH and decreased concentrations of progesterone at the time of removal of IVD's. Lesser circulating concentrations of progesterone during the period of treatment with an IVD may have been the main factor contributing to enhanced pregnancy rates to AI in heifers due to relatively low percentage of new CL's induced after administration of GnRH in heifers. Further modification to an 18‐day GnRH protocol is recommended to improve ovulation rates to the first injection of GnRH in an attempt to improve pregnancy rates to AI.

## Conflicts of interest and sources of funding

The authors declare no conflicts of interest or sources of funding for the work presented here.

## Appendix

**Table A1:** Results of logistic regression analysis of factors affecting pregnancy rates to AI and cumulative pregnancy rates 8 weeks after commencing AI in animals treated with a 7‐ or 18‐day GnRH‐based treatment with analyses separated for each animal type

Variable	Type	Covariate	df	B	SE	χ^2^	Odds ratio	CI	P‐value	Reference group
Pregnancy rate to AI	Heifers	GnRH‐7	1	−0.65	0.32	4.05	0.52	0.28–0.98	0.044	GnRH‐18
	Heifers	No CL visualised day −11	1	0.57	0.33	3.04	1.77	0.93–3.35	0.081	CL visualised day ‐ 11
	NL cows	GnRH‐7	1	−0.193	0.40	0.23	0.83	0.37–1.82	0.633	GnRH‐18
	NL cows	No CL visualised day −11	1	0.62	0.49	1.61	1.85	0.72–4.81	0.204	CL visualised day ‐ 11
	Lactating cows	GnRH‐7	1	0.24	0.29	0.67	1.27	0.72–2.25	0.412	GnRH‐18
	Lactating cows	No CL visualised day −11	1	0.13	0.32	0.18	1.14	0.61–2.14	0.675	CL visualised day −11
8‐week pregnancy rate	Heifers	GnRH‐7	1	−0.35	0.38	0.87	0.70	0.34–1.48	0.352	GnRH‐18
		2016	1	−2.01	0.48	17.37	0.13	0.05–0.34	<0.001	2018
		2017	1	−0.95	0.47	4.14	0.39	0.15–0.97	0.042	2018
	NL cows	GnRH‐7	1	−0.45	0.68	0.43	0.64	0.17–2.41	0.510	GnRH‐18
		2016	1	−0.87	0.78	1.26	0.42	0.09–1.92	0.262	2018
		2017	1	1.66	0.87	3.61	5.25	0.95–29.0	0.057	2018
	Lactating cows	GnRH‐7	1	0.70	0.33	4.58	2.02	1.06–3.83	0.032	GnRH‐18
		2016	1	−0.41	0.44	0.87	0.66	0.28–1.58	0.351	2018
		2017	1	0.07	0.46	0.02	1.07	0.44–2.63	0.883	2018

B, co‐efficient; CI, 95% confidence interval; CL, corpus luteum; df, degrees of freedom; NL, nonlactating; SE, standard error.
